# 多维方法评价全氟辛酸对人结直肠癌细胞的毒性效应

**DOI:** 10.3724/SP.J.1123.2024.05022

**Published:** 2025-01-08

**Authors:** Ruijia ZHANG, Yingshi LIN, Lanyin TU, Zitong CHEN, Weiwei ZHANG, Tiangang LUAN, Baowei CHEN

**Affiliations:** 1.海南大学生命健康学院, 海南 海口 570228; 1. School of Life and Health Sciences, Hainan University, Haikou 570228, China; 2.中山大学生命科学学院, 广东 广州 510275; 2. School of Life Sciences, Sun Yat-sen University, Guangzhou 510275, China; 3.中山大学海洋科学学院, 广东 珠海 519082; 3. School of Marine Sciences, Sun Yat-sen University, Zhuhai 519082, China; 4.中山大学人工智能学院, 广东 珠海 519082; 4. School of Artificial Intelligence, Sun Yat-sen University, Zhuhai 519082, China; 5.广州市疾病控制中心, 广东 广州 510440; 5. Guangzhou Center for Disease Control and Prevention, Guangzhou 510440, China

**Keywords:** 全氟辛酸, 人结直肠肠道毒性, 代谢紊乱, 代谢基因和通路检索软件, perfluorooctanoic acid (PFOA), human colorectal enterotoxicity, metabolic disorder, Metabolic Gene and Pathway Query software

## Abstract

全氟辛酸(PFOA)的暴露与溃疡性结肠炎的发生密切相关,但目前缺少PFOA暴露对人结直肠癌细胞(HCT116)产生毒性效应的相关分子机理研究。本研究从细胞毒性表型、细胞呼吸和代谢相关基因转录水平3个层次评价了PFOA对HCT116的毒性效应。首先,利用水溶性甲臜化合物(MTS)来评价PFOA暴露对HCT116细胞相对活性的影响;随后,利用细胞外流量分析仪对HCT116的线粒体呼吸活性进行测定;最后,用定量即时聚合酶链式反应(qPCR)对HCT116中代谢相关基因的转录水平进行检测。细胞毒性实验结果表明,与对照组相比,经高浓度(300 μmol/L)PFOA暴露48 h后,HCT116的细胞活性受到显著抑制(*p*<0.01),并在G0/G1细胞周期受到阻滞,而低浓度(30、50 μmol/L)的PFOA反而提高了细胞相对活性;低浓度(50 μmol/L)的PFOA能够促进HCT116的线粒体呼吸活性。利用自主开发的Metabolic Gene and Pathway Query检索软件和比较毒理基因组学数据库,本研究发现代谢相关基因二肽酶1(*DPEP1*)和鞘甘氨酸-1激酶(*SPHK1*)与PFOA引起的溃疡性结肠炎相关。qPCR实验结果表明,高浓度(300 μmol/L)PFOA能够显著诱导*DPEP1*和*SPHK1*的转录表达上调(约8~10倍),低浓度(50 μmol/L)PFOA未引起*DPEP1*和*SPHK1*转录表达水平的变化。本文发现细胞线粒体呼吸活性是评价低浓度PFOA干扰效应的一个敏感指标,*DPEP1*和*SPHK1*介导的细胞代谢过程可能是PFOA引起肠道毒性的潜在机制。

## Abstract (en)

While human exposure to perfluorooctanoic acid (PFOA) can lead to ulcerative colitis, the molecular mechanisms responsible for PFOA-induced intestinal toxicity are unclear. Herein, we examined the toxicity of PFOA toward human colorectal cancer cells (HCT116) from three dimensions: the cytotoxic phenotype, cell respiration, and transcription levels of metabolism-related genes. Formazan was used to assess how PFOA exposure affects HCT116-cell relative viability, after which the mitochondrial respiratory activities of these cells were determined by analyzing extracellular flux. The quantitative real-time polymerase chain reaction (qPCR) method was used to detect metabolism-related gene expression levels. The cytotoxicity assay revealed that the HCT116 showed significantly inhibited relative activities compared to those of the control when exposed to 300 μmol/L PFOA for 48 h (*p*<0.01), with most cells retained at the G0/G1 stage. In contrast, the mitochondrial respiratory activities of the HCT116 were promoted by concentrations of PFOA as low as 50 μmol/L. Two genes related to cellular metabolism (dipeptidase 1 (*DPEP1*) and sphingosine kinase 1 (*SPHK1*)) were found to be related to the PFOA-promoted formation of ulcerative colitis using our self-developed Metabolic Gene and Pathway Query software and Comparative Toxicogenomics Database (CTD). The qPCR studies revealed that *DPEP1* and *SPHK1* expression levels were enhanced by 8-10 times in HCT116 exposed to 300 μmol/L PFOA relative to the control, whereas this trend was not observed for HCT116 exposed to 50 μmol/L PFOA. Collectively, these results suggest that the respiratory activity of cellular mitochondria may serve as an index for determining the interference effects associated with PFOA and that metabolic pathways mediated by *DPEP1* and *SPHK1* may be involved in the development of PFOA-induced ulcerative colitis. Future studies should investigate the relationships between changes in metabolism-related genes (*DPEP1* and *SPHK1*) and the mitochondrial respiratory activities of intestinal cells, and verify the roles played by the *DPEP1* and *SPHK1* genes in PFOA-induced intestinal inflammation using in-vivo models.

全氟辛酸(perfluorooctanoic acid, PFOA)是全氟/多氟化合物(per- and polyfluoroalkyl substances, PFASs)家族中最常见的一种化合物,其碳链上的氢原子均被氟原子取代。PFOA具有生物累积性、持久性、长距离迁移性和毒性,2019年《斯德哥尔摩公约》将PFOA及其盐类列为持久性有机污染物,并对其加以限制或禁止使用^[1]^。目前,PFOA的排放量已明显减少,如2014年约有78.6吨PFOA被排放到渤海湾中,而2018年该区域PFOA的排放量仅有17.48吨^[2]^;但由于PFOA具有持久性和生物累积性,其在环境(如中国济南市小清河中的PFOA水平高达1.144×10^3^ μg/L)和生物(如正常儿童血浆中的PFOA水平高达39.2 ng/mL)样品中含量较高^[3,4]^。因此,对PFOA引起的毒性效应进行研究,有助于深入理解PFOA造成的环境生态风险与健康危害。

PFOA主要通过饮用水和食物进入生物体,因此消化道是PFOA接触的第一器官^[5]^。分析韩国西海岸的鱼类样品发现,PFOA可在鱼的肠道中富集,且其在肠道中的含量明显高于肝脏、鳃和鱼肉^[6]^,该结果表明肠道可能是PFOA致毒的靶器官之一。流行病学调查发现,化学厂附近居民的溃疡性结肠炎发病率与PFOA的暴露水平呈显著正相关(*p*<0.0001)^[7]^。环境水平(5.0 μg/L)的PFOA能够引起肠道细胞发生氧化损伤、炎症和细胞死亡等,同时还会造成肠道菌群的代谢紊乱^[8]^。非显著细胞毒性水平(0.36~100 μmol/L)的PFOA,能够显著促进人结肠成纤维细胞增殖(*p*<0.05),这可能与肠道炎症效应相关,同时也说明需要对PFOA的毒性效应进行全面评价^[9]^。我们课题组^[10]^的前期研究发现,PFOA暴露可以引起人结直肠腺癌上皮细胞(DLD-1)内的葡萄糖、谷氨酰胺和脂肪酸代谢重新编程。细胞代谢紊乱是PFOA致毒的一个重要特征,且中心碳代谢的代谢异常与PFOA致毒过程密切相关^[11]^,但在PFOA暴露下结直肠细胞的毒性表型与细胞代谢异常之间的关系仍有待深入研究。

本研究从细胞毒性表型、细胞呼吸和代谢相关基因转录水平3个层次来研究PFOA暴露对人结直肠癌细胞(HCT116)的毒性效应,并对细胞毒性表型与代谢相关基因转录之间的内在关系进行了分析,初步阐明了PFOA引起细胞毒性的潜在分子机制,为理解PFOA的健康危害提供了基础数据。

## 1 实验部分

### 1.1 仪器、试剂与材料

Synergy H4多功能酶标仪(美国Bio-tek公司); CytoFLEX流式细胞仪(美国Beckman Coulter公司); Seahorse XF96细胞外流量分析仪(美国Agilent公司); T100 PCR扩增仪、CFX96 Touch实时荧光定量PCR仪(美国Bio-Rad公司); Axio Observer3倒置荧光显微镜(德国Zeiss公司)。

PFOA标准品(纯度>98%)购自德国CNW Technologies公司;胎牛血清、McCoy’s 5A培养基、胰蛋白酶(含有0.25%乙二胺四乙酸(EDTA))、磷酸盐缓冲液(PBS, pH 7.2)均购自美国Gibco公司;二甲基亚砜(DMSO)、CyQUANT细胞增殖分析试剂盒(美国Thermo Fisher公司);无水乙醇(分析纯,广东光华科技有限公司); MTS细胞增殖与细胞毒性检测试剂盒、细胞周期检测试剂盒(江苏凯基生物技术股份有限公司); Seahorse XF细胞线粒体压力测试试剂盒、Seahorse XF校准液、Seahorse XF基础培养基、Seahorse XF葡萄糖溶液(100 mmol/L)、Seahorse XF丙酮酸溶液(10 mmol/L)、Seahorse XF谷氨酰胺溶液(20 mmol/L)、寡霉素(1.0 μmol/L)、羰基氰化物4-(三氟甲氧基)苯腙(FCCP)(1.0 μmol/L)和抗霉素A-鱼藤酮(0.5 μmol/L)溶液(美国Agilent公司); Eastep Super总RNA提取试剂盒(美国Promega公司); Takara cDNA合成试剂盒、TB Green Premix Ex Taq II荧光定量试剂盒(日本Takara公司)。定量即时聚合酶链式反应(qPCR)的引物由生工生物工程(上海)股份有限公司合成。

无菌平底6孔板(703002)、无菌平底96孔板(701002,中国无锡耐思生命科技股份有限公司);无菌黑色96孔板(DQWH-96,中国上海晶安生物科技有限公司);无菌Seahorse专用96孔板和探针板(美国Agilent公司)。HCT116由中山大学肿瘤防治中心黄蓬教授课题组惠赠。

### 1.2 溶液的配制

用DMSO溶解PFOA标准品,并进行稀释,配制成浓度为1 mol/L的PFOA母液,储存于-20 ℃;临用前,用细胞完全培养基(McCoy’s 5A培养基-胎牛血清(9∶1, v/v))对PFOA母液进行稀释,制备成所需浓度的PFOA培养液。

细胞实验中用到的0.01%DMSO培养液均为使用细胞完全培养基稀释得到。

### 1.3 细胞毒性评价

用细胞完全培养基配制细胞含量为5×10^4^个/mL的HCT116悬浮液,将HCT116以5×10^3^个/100 μL的孔密度接种在平底96孔板中,培养过夜,待细胞贴壁后,用PBS(pH 7.2)清洗细胞2次,用100 μL不同浓度(1~600 μmol/L)的PFOA培养液分别处理细胞24 h和48 h,其中以0.01% DMSO培养液处理组作为阴性对照组,所有处理组均设置4个复孔;本实验还设置了空白对照组,即仅在孔板中加入相同体积的完全培养基。将10 μL水溶性甲臜化合物(MTS)加入至每个孔中,随后在37 ℃、避光的细胞培养箱中孵育3~4 h;最后,利用多功能酶标仪在490 nm波长下测量吸光度(optical density, OD)。细胞相对活性的计算公式如下:


(1) $$\text{ Cell relative activity }=\frac{{OD}_{\text{treatment group }}-{OD}_{\text{blank }}}{{OD}_{\text{negative control }}-{OD}_{\text{blank }}}\times 100\%$$

其中,O ${D_{treatment}}_{group}$ 、OD_negative control_、OD_blank_分别代表实验组、阴性对照组和空白对照组的吸光度。此外,将HCT116以8×10^3^个/100 μL的孔密度接种在黑色96孔板中,培养过夜,用0.01% DMSO培养液或不同浓度(50、100和300 μmol/L)的PFOA培养液处理细胞48 h,之后根据CyQUANT细胞增殖分析试剂盒的操作说明,向每个孔中加入100 μL的检测试剂,在37 ℃下避光孵育60 min,随后用倒置荧光显微镜的FITC通道检测细胞样品的荧光信号。

### 1.4 细胞周期分析

用细胞完全培养基配制细胞含量为2×10^5^个/mL的HCT116悬浮液,将HCT116以2×10^5^个/2 mL的孔密度接种在6孔板中,培养过夜;用0.01% DMSO培养液或不同浓度(100、300 μmol/L)的PFOA培养液处理细胞48 h,每个组设置3个复孔;之后用胰蛋白酶处理细胞,在200 g下离心5 min,收集细胞,用预冷的70%乙醇水溶液固定细胞2 h;用预冷的PBS(pH 7.2)清洗细胞2次,加入500 μL细胞周期检测试剂盒中的染色试剂(核糖核酸酶(RNase)溶液-碘化丙啶(PI)溶液(1∶9, v/v)),避光孵育30 min;最后利用CytoFLEX流式细胞仪FL2通道检测荧光信号,用ModFit软件计算细胞的周期分布。

### 1.5 线粒体氧化应激测试

用细胞完全培养基配制细胞含量为1×10^5^个/mL的HCT116悬浮液,将HCT116以1×10^4^个/100 μL的孔密度接种于Seahorse专用96孔板中,培养过夜;用0.01% DMSO培养液或不同浓度(50、100 μmol/L)的PFOA培养液处理细胞48 h,每组设置5个复孔。将97 mL Seahorse XF基础培养基、1 mL Seahorse XF葡萄糖溶液、1 mL Seahorse XF丙酮酸溶液和1 mL Seahorse XF谷氨酰胺溶液混合,制备Seahorse检测培养液(pH 7.4)。用Seahorse检测培养液(pH 7.4)清洗细胞2次后,再加入180 μL Seahorse检测培养液,随后将孔板置于37 ℃且无二氧化碳的培养箱中平衡40~60 min。将200 μL Seahorse XF校准液加入到探针板中,在37 ℃下孵育过夜;分别将Seahorse XF细胞线粒体压力测试试剂盒中的20 μL寡霉素(1.0 μmol/L)、22 μL FCCP(1.0 μmol/L)和25 μL抗霉素A-鱼藤酮(0.5 μmol/L)溶液加入到上述探针板的A、B和C孔中,随后将探针板放入到Seahorse XF96细胞外流量分析仪中并进行校准,最后对载有细胞的Seahorse专用96孔板进行检测。利用Seahorse XF96细胞外流量分析仪测量活细胞的有氧消耗速率(oxygen consumption rate, OCR);同时对每个孔细胞中的蛋白质含量进行检测,并利用蛋白质含量对OCR进行标准化处理,用Wave软件处理数据。

### 1.6 代谢基因和通路的检索

基于京都基因和基因组(Kyoto Encyclopedia of Genes and Genomes, KEGG)数据库,通过KEGG API获取代谢通路上的所有基因列表(http://rest.kegg.jp/get/hsa00020),收集KEGG数据库中人类相关的代谢基因和代谢通路。基于Reactome数据库,通过Pathway Browser查看及下载靶向代谢通路相关功能基因的所有信息(https://reactome.org/PathwayBrowser/#/R-HSA-71403&DTAB=MT)。对由KEGG和Reactome数据库下载的参考基因和代谢通路数据进行整合并去冗余,建立人类相关代谢通路及功能基因的数据库,再利用自主开发的Metabolic Gene and Pathway Query软件,实现人类相关代谢基因和通路的快速检索。利用Metabolic Gene and Pathway Query软件,根据代谢基因对其所涉及的代谢通路进行检索,并链接到KEGG数据库以查看详细信息;同时,根据代谢通路可以查看所有的代谢基因,并链接到PubMed Gene数据库以查阅更详细信息。

### 1.7 qPCR实验

用细胞完全培养基配制细胞含量为2×10^5^个/mL的HCT116悬浮液,将HCT116以2×10^5^个/2 mL的孔密度接种在6孔板中,培养过夜;用0.01% DMSO培养液或不同浓度(50、100和300 μmol/L)的PFOA培养液处理细胞48 h,每组设置4个复孔。采用Eastep Super总RNA提取试剂盒提取细胞中的总RNA,利用多功能酶标仪测量RNA的浓度和纯度。依据Takara cDNA合成试剂盒说明,配制含有1.0 μg RNA的反转录混合溶液,用T100 PCR扩增仪对上述提取的RNA进行反转录,反应条件为37 ℃和15 min,最终获得DNA模板。将TB-Green Premix Ex Taq II荧光试剂、引物溶液(正向引物和反向引物,具体信息见表1)和DNA模板加入至25 μL qPCR反应体系中,用实时荧光定量PCR仪进行实时荧光信号检测,并测量每次运行的溶解曲线。以*GAPDH*为管家基因,采用Livak法计算二肽酶1(*DPEP1*)基因和鞘甘氨酸-1激酶(*SPHK1*)基因的相对表达量^[10]^,其中*DPEP1*和*SPHK1*的引物序列信息由PrimerBank数据库中查询获得。

**表1:** qPCR引物信息

Gene	5'-3' forward	5'-3' reverse
*DPEP1*	CAAGTGGCCGACCATCTGG	GGGACCCTTGGAACACCATC
*SPHK1*	GCTCTGGTGGTCATGTCTGG	CACAGCAATAGCGTGCAGT
*GAPDH*	GCACCGTCAAGGCTGAGAAC	TGGTGAAGAACGCCAGTGGA

### 1.8 统计分析

使用单因素方差分析法来评价细胞相对活性、细胞周期分布比例、线粒体OCR及基因转录表达水平在PFOA处理组和阴性对照组之间的差异。仅当单因素方差分析存在统计学差异时,对两两组间进行统计学分析。当出现方差齐性时,采用Bonferroni试验对两两组间进行比较;当出现方差不齐时,采用Dunnett’s T3 post hoc试验对两两组间进行比较,其中显著性水平设为*p*<0.05。

## 2 结果与讨论

### 2.1 PFOA暴露对HCT116毒性表型的影响

在暴露于300 μmol/L PFOA 24 h后,HCT116的细胞相对活性并未产生显著变化(*p*>0.05),如图1a所示;当暴露时间延长至48 h, 300 μmol/L的PFOA能够抑制约20%的细胞相对活性(*p*<0.01),而低浓度(30、50 μmol/L)的PFOA反而提高了细胞相对活性(图1b)。有研究报道,随着PFOA暴露时间的增加,HCT116的细胞活性会逐渐受到抑制,如经PFOA暴露4 h后的半数效应浓度(EC_50_)值为1.32 mmol/L,暴露24 h后的EC_50_值为0.94 mmol/L,暴露72 h后的EC_50_值为0.31 mmol/L^[12]^。先前研究表明,经300 μmol/L PFOA暴露48 h,DLD-1的细胞活性约损失20%^[10]^,与本文结果一致。此外,有研究^[13]^表明,当PFOA的暴露浓度高于200 μmol/L时,人乳腺上皮细胞的增殖会受到抑制,但50 μmol/L的PFOA可诱导人乳腺上皮细胞的增殖。因此,不同的PFOA暴露浓度可能引起截然不同的毒性效应,即抑制效应或诱导效应。

**图1:**
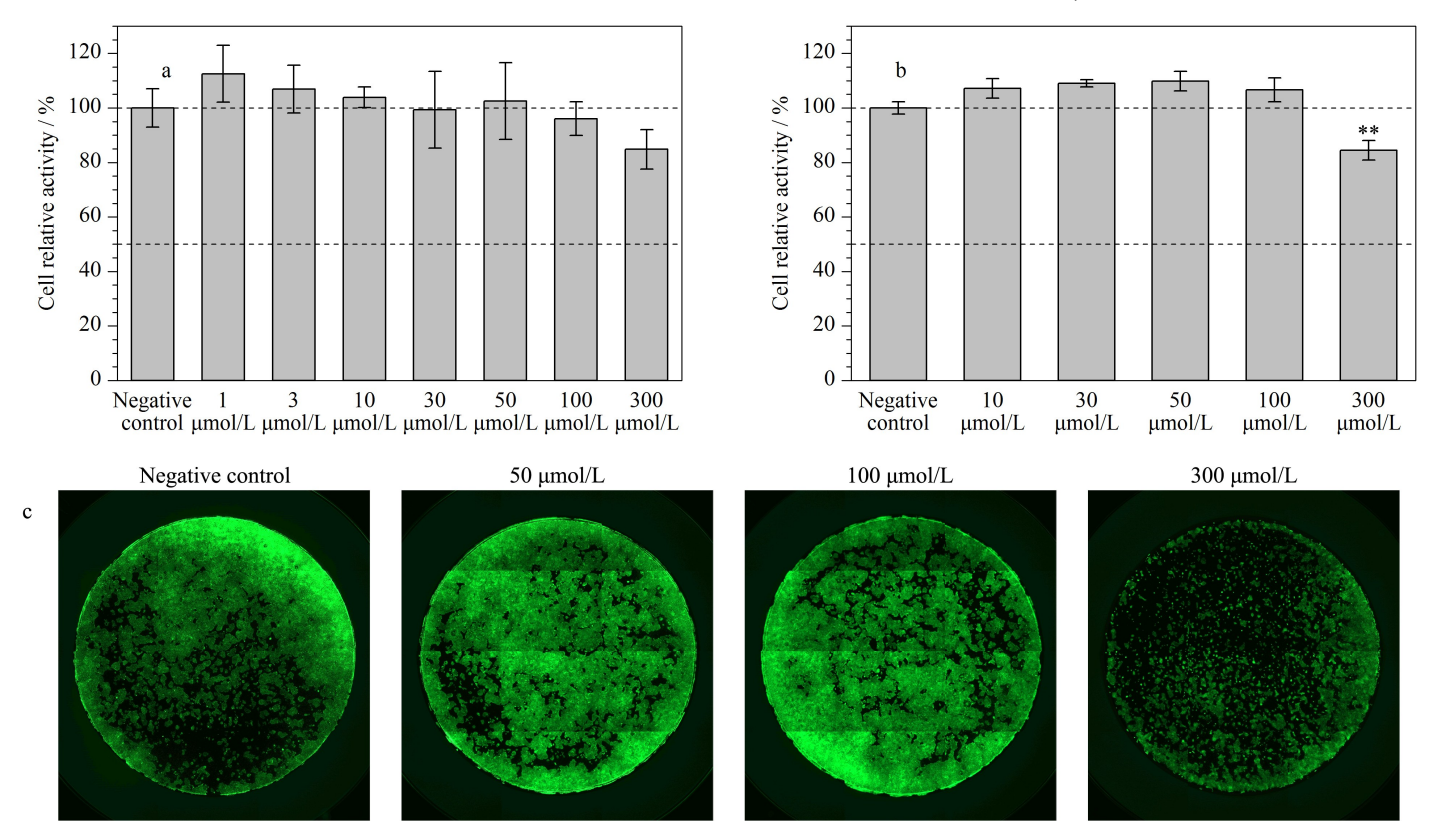
HCT116经不同浓度PFOA处理(a)24 h和(b)48 h后的细胞相对活性(*n*=4), (c) HCT116经不同浓度PFOA处理48 h后的活细胞荧光成像图

活细胞荧光成像结果显示,经PFOA处理48 h后,随着PFOA的暴露浓度增加至300 μmol/L, HCT116的荧光信号强度明显下降,说明存活率明显下降(图1c),再次证明了高浓度(300 μmol/L)的PFOA能够显著引起HCT116的细胞毒性。PFOA暴露下的HCT116细胞周期分布比例如图2所示。结果表明,与阴性对照组相比,100 μmol/L的PFOA不会影响HCT116的细胞周期,但当暴露浓度达到300 μmol/L时,处于G0/G1期的HCT116平均比例为84.10%,显著高于阴性对照组(49.91%)和100 μmol/L PFOA处理组(51.47%)(*p*<0.01),说明HCT116在G0/G1期受到阻滞,即细胞分裂周期被延长、细胞增殖被抑制,这也与图1a~c的结果一致。

**图2:**
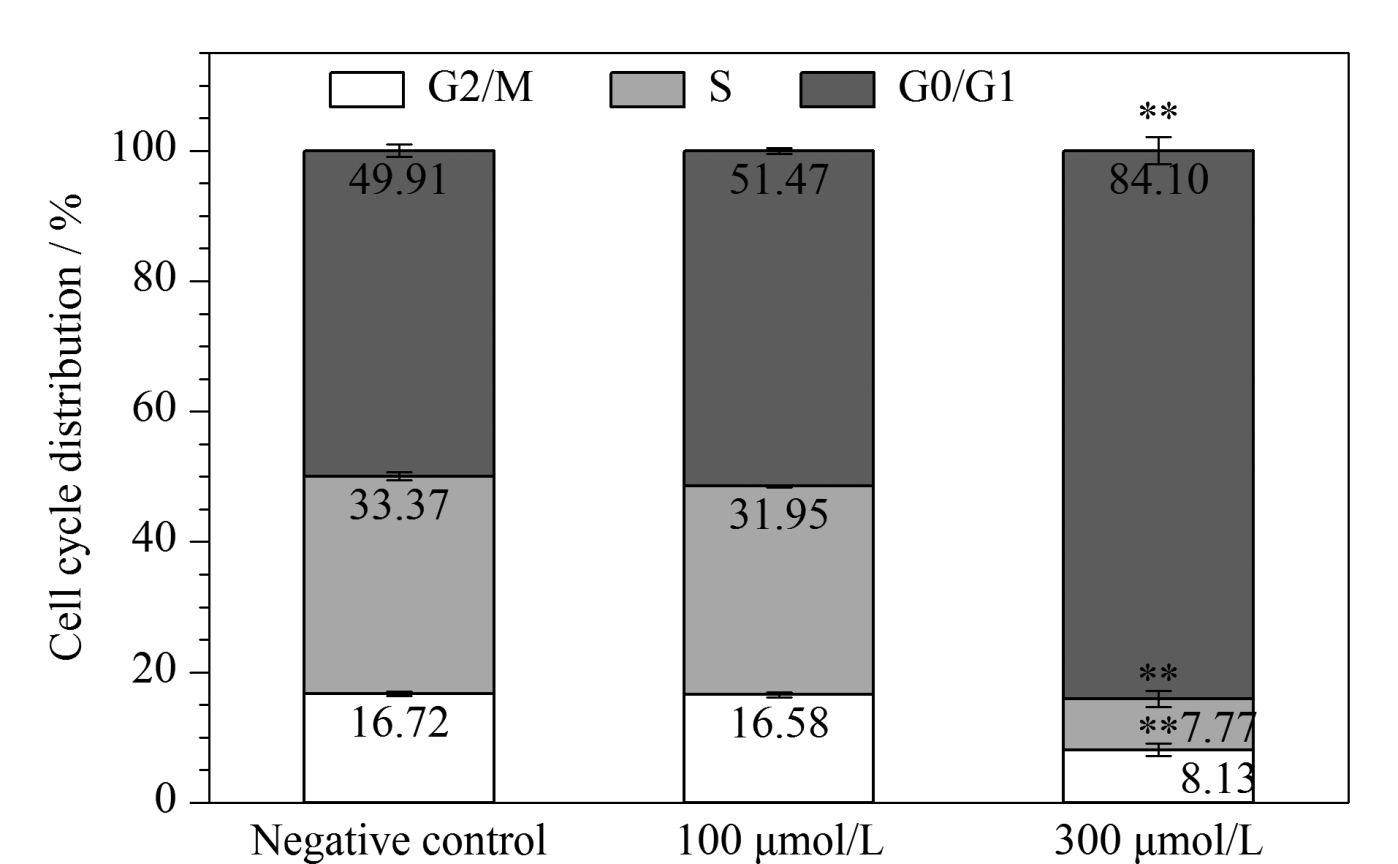
HCT116经不同浓度PFOA处理48 h后的细胞周期分布(*n*=3)

上述结果说明,PFOA能够扰乱细胞周期,这可能与其所抑制的细胞活性相关。

有研究^[14]^报道,经300 μmol/L PFOA暴露24、48或72 h后,DLD-1在G0/G1期的比例均得到了显著增加(*p*<0.05),而100 μmol/L的PFOA没有对DLD-1的细胞周期分布产生干扰。总之,当PFOA的暴露浓度达到300 μmol/L且暴露时间为48 h时,其会抑制HCT116的细胞相对活性,并干扰细胞周期。

### 2.2 PFOA暴露对HCT116线粒体呼吸活性的影响

线粒体是细胞内三磷酸腺苷(ATP)的生产工厂,并且线粒体呼吸活性与能量代谢密切相关。实验考察了50 μmol/L和100 μmol/L PFOA对HCT116线粒体呼吸活性的影响。实验结果表明,在对细胞数量作均一化处理后,50 μmol/L PFOA处理组中HCT116的平均OCR为4.1×10^3^~2.6×10^4^ pmol/(min·mg protein),而100 μmol/L PFOA处理组中HCT116的平均OCR(3.8×10^3^~2.4×10^4^ pmol/(min·mg protein))与阴性对照组(3.2×10^3^~2.3×10^4^ pmol/(min·mg protein))接近(图3a)。与阴性对照组相比,在50 μmol/L PFOA暴露下,HCT116的基础OCR(图3b)、ATP合成相关OCR(图3c)、细胞最大呼吸速率(图3d)和细胞备用呼吸能力(图3e)均显著提高(*p*<0.05),而非线粒体呼吸耗氧量未改变(图3f)。研究报道,250 μmol/L和300 μmol/L的PFOA能够分别抑制人神经细胞和人肝细胞的线粒体呼吸活性,表明高浓度的PFOA在抑制细胞相对活性的同时也会损伤线粒体的呼吸功能^[13,15,16]^。还有研究^[16]^报道,100 μmol/L的PFOA未对人肝细胞的细胞相对活性和线粒体呼吸活性产生影响,这也与本文的研究结果一致。先前的动物暴露实验发现,PFOA能够激活大鼠的Pgc-1*α*信号通路,诱导线粒体呼吸链相关酶的基因转录表达,促进线粒体呼吸^[17]^。本研究发现,与阴性对照组相比,50 μmol/L的PFOA能够促进HCT116的线粒体呼吸活性,这可能是因为PFOA激活了PGC-1*α*信号通路,表明细胞线粒体呼吸活性是评价低浓度PFOA干扰效应的一个敏感指标。

**图3:**
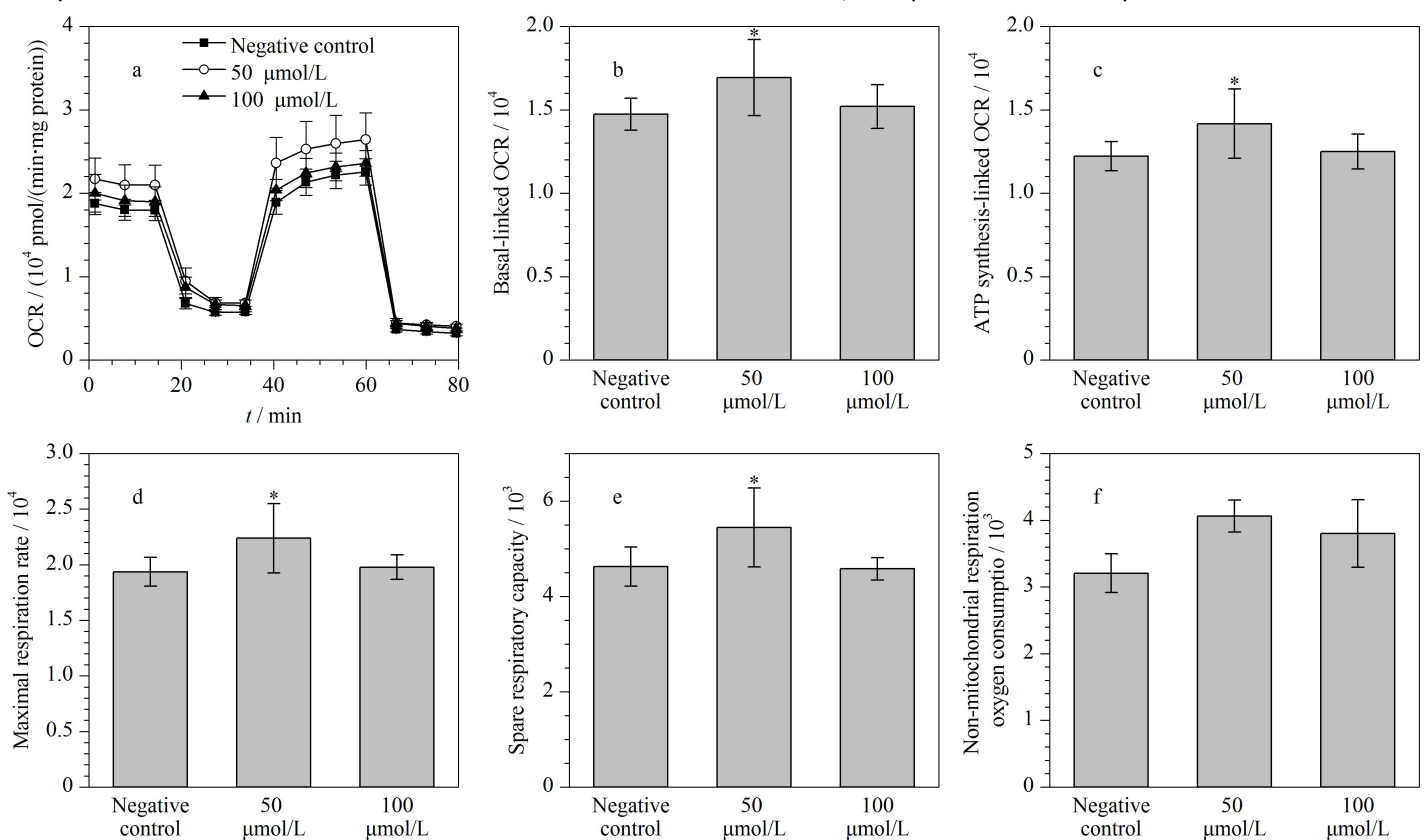
不同浓度的PFOA对HCT116的(a)OCR、(b)基础OCR、(c)ATP合成相关OCR、(d)最大呼吸速率、(e)备用呼吸能力及(f)非线粒体呼吸耗氧量的影响(*n*=5)

### 2.3 PFOA暴露对HCT116代谢基因转录表达的影响

基于比较毒理基因组学数据库(CTD, http://ctdbase.org/),对PFOA暴露与人类肠道疾病之间的关联性进行分析。实验结果表明,PFOA暴露与溃疡性结肠炎(ulcerative colitis, UC)存在相关性,共涉及30个不同的功能基因(*ALB*、*APOA4*、*C3*、*CASP3*、*CDH1*、*CFB*、*CXCL8*、*DAP*、*DPEP1*、*FCGR2A*、*HERC2*、*HLA-DRB1*、*HNF4A*、*HP*、*IL10*、*IL12B*、*IL1B*、*IL7R*、*JAK2*、*KIF21B*、*LTF*、*MMP9*、*MPO*、*MST1*、*RELA*、*SLC11A1*、*SPHK1*、*STAT3*、*TNF*、*TNFSF15*)。使用Metabolic Gene and Pathway Query软件对这30个功能基因与代谢通路之间的关系进行分析,结果发现*DPEP1*基因和*SPHK1*基因分别与脂肪酸生物合成和鞘磷脂代谢通路相关。为进一步研究PFOA对HCT116代谢过程的影响,分别用不同浓度(50、100、300 μmol/L)的PFOA处理HCT116 48 h,用实时荧光定量PCR仪检测细胞内*DPEP1*基因和*SPHK1*基因的转录表达水平。结果显示,与阴性对照组相比,高浓度(300 μmol/L)PFOA处理组中的*DPEP1*基因和*SPHK1*基因相对表达水平上调约8~10倍,而低浓度(50、100 μmol/L)PFOA暴露没有改变*DPEP1*基因和*SPHK1*基因的表达水平(图4)。

**图4:**
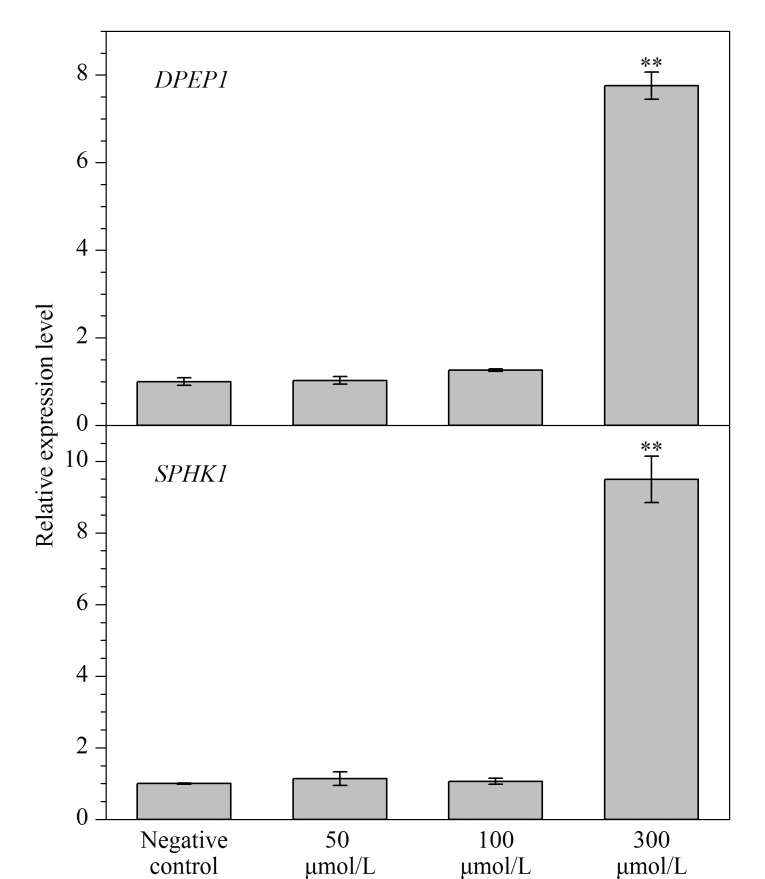
HCT116经不同浓度PFOA处理48 h后*DPEP1*基因和*SPHK1*基因的相对表达水平(*n*=4)

*DPEP1*基因可通过催化反应将白三烯D4转化为白三烯E4,从而调节白三烯的活性,而白三烯作为炎症因子可以促进炎症反应^[18]^。*SPHK1*基因可催化鞘氨醇的磷酸化,从而形成鞘氨醇-1-磷酸(S1P), S1P能够激活TNF-*α*和NF-*κ*B信号通路,其在炎症过程中发挥着重要作用^[19]^。人群流调发现,溃疡性结肠炎与*DPEP1*基因和*SPHK1*基因表达上调显著相关^[20,21]^。蛋白质组分析表明,DPEP1蛋白介导了双酚A引起的溃疡性结肠炎^[22]^。动物实验发现,2,4,6-三硝基苯磺酸钠通过激活sphk/S1P信号通路可引起溃疡性结肠炎^[23]^。上述研究结果表明,外源性物质诱导的*DPEP1*基因和*SPHK1*基因高表达与溃疡性结肠炎的发生密切相关,本研究中高浓度(300 μmol/L)的PFOA显著提高了*DPEP1*基因和*SPHK1*基因的表达水平,这可能是PFOA引起肠道毒性的原因之一,同时这也与HCT116的毒性表型结果一致。

## 3 结论

本研究从细胞毒性表型、细胞呼吸和代谢相关基因转录水平3个层次评价了PFOA对HCT116的毒性效应。在高浓度(300 μmol/L)PFOA暴露条件下,HCT116的*DPEP1*基因和*SPHK1*基因表达水平上调,这可能是PFOA引起肠道细胞毒性的潜在分子机制;与此同时,低浓度(50 μmol/L)的PFOA就能够引起HCT116线粒体呼吸活性的明显变化,因此细胞线粒体呼吸活性可作为评价低浓度PFOA干扰效应的敏感指标。未来可对代谢相关基因(*DPEP1*和*SPHK1*)改变与肠道细胞线粒体呼吸活性之间的关系进行深入研究,并利用体内模型验证*DPEP1*基因和*SPHK1*基因在PFOA诱导肠道炎症中的作用。
